# Der (Un)Sinn von Purpose: Theoriebasierte Ansätze zur Gestaltung von sinnhaftem Handeln in Unternehmen

**DOI:** 10.1007/s11612-022-00628-7

**Published:** 2022-04-19

**Authors:** Stefanie Krügl

**Affiliations:** Fachhochschule Südwestfalen, Iserlohn, Deutschland

**Keywords:** Purpose, Vision, Sinnorientierte Arbeitsgestaltung, Wertebasierte Führung, Purpose, Vision, Meaningful work design, Value-based leadership

## Abstract

Dieser Beitrag der Zeitschrift „Gruppe. Interaktion. Organisation.“ beschäftigt sich mit der Frage nach dem (Un‑)Sinn von Purpose. Drei Dinge fehlen den aktuellen Ansätzen zur Gestaltung kollektiver Sinnhaftigkeit und Ausrichtung von Arbeit auf den Unternehmenszweck – gemeinhin bekannt als „Purpose“: Orientierung (*was* mit Purpose gemeint ist), Klarheit (*wie* Purpose zu gestalten ist) und Evidenz (*warum* Purpose gestaltet werden sollte) – kurz: das What, How und Why von Purpose. Der vorliegende Beitrag schließt anhand der Betrachtung des aktuellen Literaturstands diese drei Lücken. Purpose wird theoriebasiert definiert (What). Es werden drei Ebenen skizziert, auf denen Purpose adressiert werden sollte: Individuum, Team und Organisation. Dazu werden wissenschaftlich untersuchte Stellschrauben erläutert und Hinweise gegeben, wie Unternehmen Arbeit sinnhaft gestalten sollten (How). Da sich die bisherige Diskussion zum „Sinn des Purpose“ kaum äußert, werden die Gründe beleuchtet, die dafürsprechen, sich als Unternehmen und Führungskraft mit diesem – in sinnvoller Weise – zu beschäftigen (Why).

## Abstract (en)

This contribution to the journal “Group. Interaction. Organization.” deals with the question of the (non-)sense of purpose. Current approaches aiming at shaping the collective meaningfulness and aligning employee actions with the company’s purpose miss three crucial points: Orientation (Meaning of purpose), clarity (How to shape purpose?), and evidence (Why address purpose in organizations?). In short: The *What, How* and *Why *of purpose. This contribution fills these three gaps by providing a comprehensive review of literature. Purpose is defined (*what*) in a theory-driven way. The three levels at which purpose should be addressed (individual, team, and organization) are outlined. Relevant insights on these levels and advice for meaningful work design are provided (*how*). As current ongoing discussions barely contribute on the sense of purpose as such, the intention of this article is to shed light on why companies and managers should engage with this subject in a meaningful way (*why*).

## Einleitung

Unternehmen von heute brauchen einen „Purpose“, sagen einhellig all die „Leadership Gurus“, „Innovation Evangelists“ und „Corporate Rebels“. In Zeiten, in denen nur noch der Wandel stabil scheint, verwundert es kaum, dass die tief menschliche Sehnsucht nach einem Sinn im Leben (Frankl 1959) eine Renaissance erlebt. Unternehmen streben nach einer starken gemeinsamen Ausrichtung, auf die Mitarbeitende mit voller Kraft hinarbeiten. Der Unternehmenserfolg soll automatisch folgen. Viele der aktuell in Unternehmen eingeführten Ansätze für Purpose degenerieren im Zuge dieses Trends zu Modevokabeln (Backovic 2021). Was Purpose bedeutet und wie er zu gestalten ist, ist häufig kaum mit validen Fakten untermauert, stattdessen unscharf bis transzendental konnotiert – bis hin zu „Visionen, die ein organisationales Jenseits“ suggerieren (Lackner und Schuster 2020, S. 405).

Bedauerlicherweise entwertet der marktgetriebene Purpose-Hype ein prinzipiell wertvolles Instrument der Organisationsentwicklung, weil es der aktuell geführten Diskussion an drei Dingen fehlt: Orientierung, Klarheit und Evidenz. Die Diskussion ist orientierungslos, weil es infolge kaum belastbarer Definitionen an greifbaren Gegenständen mangelt. *Was* ist mit Purpose genau gemeint? Die Diskussion ist konfus, weil Ansatzpunkte zur Entwicklung einer Grundausrichtung im Sinne eines Purpose weitgehend unklar bleiben. *Wie* kann man Purpose tatsächlich entwickeln? Die aktuelle Diskussion ist zudem hoch spekulativ, weil sich die behaupteten Effekte eines Purpose in der wissenschaftlichen Literatur nicht nachweisen lassen bzw. nachgewiesene Effekte außer Acht gelassen werden: *Warum* also sollten sich Unternehmen mit Purpose beschäftigen? Der Gestaltung von Purpose in Unternehmen fehlt es ausgerechnet an Simon Sineks zentralen Stellschrauben des *What, How* und *Why *(Sinek 2019). Darin besteht der aktuell vorherrschende Un-Sinn von Purpose.

Mit dem vorliegenden Beitrag wird theoriegeleitet Klarheit in das diffuse Konstrukt Purpose und dessen Gestaltung gebracht. Dabei orientiert sich der Artikel am eingeführten Dreiklang aus *What, How* und *Why*. Erstens wird Purpose sauber und fundiert abgegrenzt. Zweitens werden sechs Wirkprinzipien beschrieben, mit denen Purpose auf den Ebenen Individuum, Team und Unternehmen adressiert werden kann. Drittens wird dargestellt, welche Gründe dafürsprechen, sich als Unternehmen tatsächlich sinnhaft mit Purpose auseinanderzusetzen.

## Was ist Purpose?

Der Begriff des Purpose wird häufig, basierend auf der Beschreibung von (Ryff 1989b, S. 43/44), als Zuschreibung von Richtung, Intentionalität und Integration der verschiedenen Lebensbereiche definiert. Vermutlich macht dieses Verständnis von Purpose als handlungsleitende Grundausrichtung das Konzept für Unternehmen so attraktiv. In der Verwendung von Ryffs Definition wird gerne die Tatsache unterschlagen, dass sie Purpose aus persönlichkeitspsychologischer Sicht als Kennzeichen einer intakten psychischen Gesundheit definiert, als „persönliche Reife, die ein klares Verständnis des eigenen Lebenssinns, ein Gefühl von Zielgerichtetheit und Intentionalität umfasst“ (Ryff 1989a, S. 1071). Entsprechend ist ein sinnhafter Einsatz individueller Ressourcen und Stärken zunächst einmal stark personengebunden und subjektiv.

Rosso et al. (2010, S. 108) betrachten Purpose als einen von sieben Faktoren, die dazu führen, dass Arbeit als sinnvoll wahrgenommen wird^1^. Dabei unterscheiden sie zwischen zwei Arten, Purpose zu verstehen: als individuelle Bedeutsamkeit einerseits und als geteilte Wertvorstellungen einer Gemeinschaft, z. B. Team oder Organisation, andererseits (Rosso et al. 2010, S. 111).

Erstens entsteht Purpose für jeden Menschen individuell durch die wahrgenommene Bedeutsamkeit der eigenen Aufgaben (Rosso et al. 2010). Grant (2008) zeigte etwa, dass Mitarbeitende, die den sozialen Nutzen ihrer Arbeit als hoch einstufen, diese als sinnhaft wahrnehmen. Ferner erzielen diese Mitarbeitenden, deren Arbeit mit prosozialem Sinn aufgeladen ist, bessere Leistungen (Grant 2008). Ryff (1989a) betonte in ihrer Definition zudem die temporäre Dimension des sich im Zuge äußerer oder persönlicher Veränderungen wandelnden individuellen Purpose. Jiang (2021) zeigte in einer Längsschnittstudie, dass Menschen unter Krisenbedingungen den Purpose ihrer Arbeit an die jeweiligen Gegebenheiten anpassen. Mitarbeitende einer US-amerikanischen Flüchtlingsbehörde waren während der syrischen Flüchtlingskrise über zwei Jahre immensen Veränderungen in ihrer Arbeit ausgesetzt. Die Mitarbeitenden hielten am grundlegenden Sinn ihrer Arbeit fest, definierten aber in der geänderten Situation ein vorläufiges neues „Warum“ ihrer Arbeit und fokussierten sich weniger auf Qualität, sondern mehr auf Quantität, d. h. die schnelle Bearbeitung von Asylanträgen. Dass der individuelle Purpose auch über eine Lebensspanne stabil sein kann, zeigen umfassende Forschungsarbeiten zum Thema der persönlichen Berufung (s. unten) (z. B. Dobrow und Tosti-Kharas 2011; Dobrow und Heller 2015).

Zweitens entsteht Purpose in Bezug auf den Beruf durch ein von einer Gruppe geteiltes Wertesystem, insbesondere in einer Gesellschaft, in welcher Arbeit eine wesentliche Grundlage für Selbstwert darstellt (Baumeister 1991). So verstanden bedeutet Purpose eine Art sozialen Kompass für richtig und falsch, der die menschliche Wahrnehmung und das Verhalten leitet (Schwartz 1992). Ein Handeln in Übereinstimmung mit dem gemeinsamen Wertesystem gibt dem Individuum die Gewissheit, „das Richtige getan zu haben“ (Baumeister und Vohs 2002, S. 610), und erzeugt ein Gefühl für zielgerichtetes Handeln (Frankl 1959). Purpose bildet laut Baumeister (1991, S. 118) die Grundlage, warum Mitarbeitende ihre Arbeit machen, und warum diese für sie wertvoll, wichtig und wert ist, getan zu werden. Traditionell waren mit Arbeit verbundene individuelle Ziele eher kongruent mit Unternehmenszielen, etwa Gewinn- und Statusmaximierung. Die Nachwuchsgenerationen dagegen vertreten Werte wie Sinnstiftung, Arbeitszufriedenheit und Selbstverwirklichung und stellen damit neuartige Forderungen an das Wertesystem von Organisationen (Lancaster und Stillman 2010). So führen sie Organisationen nicht nur zurück an die Wurzel ihrer Existenz, die neben dem Wunsch nach finanziellem Erfolg stets eine qualitative Komponente enthält (van Knippenberg 2020), sondern bringen sie auch dazu, sich an äußeren, gesellschaftlichen Werten und Veränderungen auszurichten, um Arbeit sinnstiftend und attraktiv zu gestalten (Grant 2008).

Häufig wird Purpose in einem Atemzug mit der Vision bzw. Mission einer Organisation genannt. Van Knippenberg (2020, S. 7) grenzt Vision und Purpose klar gegeneinander ab und beschreibt, in Anlehnung an Selznick (1957), Unternehmenspurpose als Verständnis dafür, was der grundlegende Zweck der Organisation ist und warum dieser sinnvoll sei. Der Zweck sei immer gegenwartsgerichtet und obligatorisch vorhanden – jede Organisation muss in ihrem Tun einen Zweck verfolgen, während eine Vision als vorgestelltes Zukunftsbild auch optional sein kann.

Stellen die Vision das Zukunftsbild und die Mission grundlegende Ziele, Werte und Zwecke einer Organisation dar (Rosso et al. 2010), so ist Purpose als „Richtung und Intentionalität“ (Ryff 1989a, S. 1071) sozusagen die dritte Komponente erfolgreicher Organisationsentwicklung, der „Motor“, der das aktive Handeln im Sinne der Vision und Mission antreibt. Ein gemeinsamer Purpose entsteht entsprechend, wenn mehrere Mitarbeitende das gleiche Verständnis vom Sinn und Zweck ihrer Arbeit teilen (Carton et al. 2014, S. 1544).

Entflechtet man also den Begriff des Purpose, wird deutlich, dass mehrere Ebenen unterschieden werden müssen: der individuelle Purpose als persönliche Sinnfindung bzw. Berufung und der Purpose einer Gemeinschaft, sprich eines Teams von Menschen, als geteiltes Wertesystem und übergeordnet einer Organisation als Unternehmenszweck, auf den sich die Aktivitäten der Gemeinschaft ausrichten.

## Wie lässt sich Purpose entwickeln?

**Figure 1:**
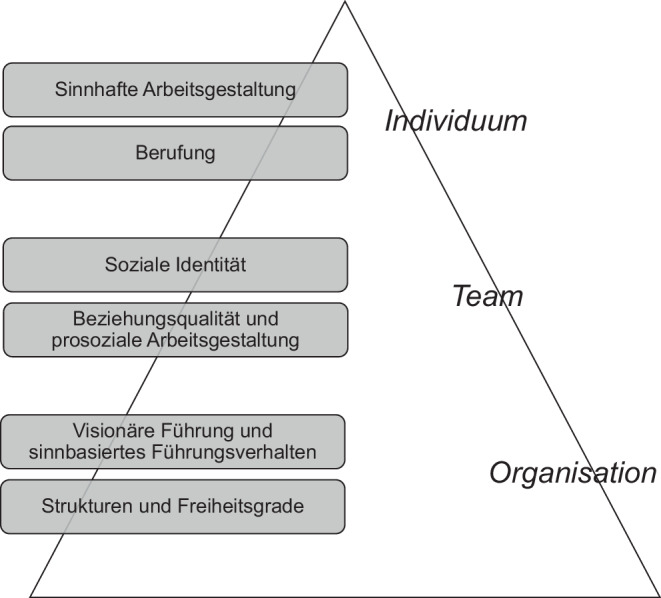

Um das Handeln von Menschen sinnstiftend zu gestalten und damit Purpose zu erzeugen, können Unternehmen an sechs substanziellen Wirkmechanismen auf drei Ebenen ansetzen:* Individuum, Team* und *Organisation* (siehe Abb. 1). Ein Individuum kann sich mit dem Unternehmenszweck identifizieren, wenn Arbeit sinnhaft gestaltet ist. In manchen Fällen kann dies bis zur Verwirklichung einer individuellen Berufung in der Arbeitstätigkeit führen, wie etwa als Musiker:in. Auf Teamebene kann Purpose an der sozialen Identität sowie Beziehungsqualität und prosozialer Arbeitsgestaltung ansetzen. Die Arbeit am Purpose der Organisation liegt in den Feldern visionäre Führung bzw. sinnbasiertes Führungshandeln sowie dem Herstellen eines Gleichgewichts von Strukturen und Freiheitsgraden.

### Individuum: Purpose als subjektive Sinnwahrnehmung

#### Sinnhafte Arbeitsgestaltung und Persönlichkeit

Sinnstiftende Arbeit ist mit hoher persönlicher Relevanz, positiver Bedeutung (Rosso et al. 2010) und folglich höherer Arbeitsmotivation verbunden (Grant 2008). Grant (2008) wies die Rolle der Bedeutsamkeit einer Tätigkeit für Mitarbeitende zur Steigerung der Arbeitsleistung nach. In drei Feldexperimenten untersuchte er den Leistungsunterschied zwischen Callcenter-Mitarbeitenden im Fundraising, von denen einigen (der Experimentalgruppe) der Sinn ihrer Tätigkeit vermittelt wurde, während die anderen keine Information darüber erhielten. Es überrascht kaum, dass die Experimentalgruppe signifikant bessere Ergebnisse erzielte. Diese Erkenntnisse verdeutlichen, dass Mitarbeitende deutlich bessere Leistungen erbringen, wenn sie verstehen, welchen Sinn ihre Aufgabe hat. Welche Aktivitäten aber Sinn stiften, ist im Kern eine individuelle Entscheidung.

Laut der Theorie der sinnorientierten Arbeitsgestaltung nach Barrick et al. (2013) führt individuelle Wahrnehmung dazu, dass Menschen motiviert sind und entsprechend handeln, wenn sie glauben, dass ihr Handeln zum Ziel führen kann (*Zweckmäßigkeit*), und sie ihr Handeln als bedeutsam empfinden (*Sinnhaftigkeit*). Dabei bringen selbstregulatorische Prozesse Merkmale der Persönlichkeit und der Situation in Einklang. Menschen verhalten sich zielgerichtet und motiviert, wenn eine als zielführend und sinnhaft wahrgenommene Arbeitstätigkeit Verhaltensweisen eines jeweils individuell übergeordneten Motivs aktiviert (Gemeinschaft, Status, Leistung oder Autonomie). Persönlichkeitsmerkmale (Extraversion, emotionale Stabilität, Offenheit, Verträglichkeit und Gewissenhaftigkeit) beeinflussen, wie stark und häufig diese Verhaltensweisen ausgeübt werden (Williamson Smith und DeNunzio 2020). Demnach treibt offene Menschen das Bedürfnis nach Autonomie an, was dazu führt, dass sie Tätigkeiten mit einem hohen Maß an Gestaltungsspielraum und Aufgabenvielfalt bevorzugen. Für gewissenhafte Personen ist es dagegen wichtig, fleißig zu sein und Anerkennung zu erhalten. Solchen Menschen hilft häufiges Feedback dabei, motiviert zu arbeiten, eine starke Leistung zu zeigen und ihre Arbeit als bedeutsam zu empfinden.

Führungskräfte können diese Erkenntnisse für die Zuordnung von Aufgabenbereichen für ihre Mitarbeitenden nutzen. Indem sie in regelmäßigen Mitarbeitergesprächen die erlebte Motivation in Verbindung mit Arbeitsmerkmalen und dem, was sich Mitarbeitende wünschen, vergleichen, ergründen Mitarbeitende und Führungskräfte gemeinsam den Weg der besten Entwicklung des Mitarbeitenden. Unternehmen tun gut daran, die individuell sinnstiftenden Faktoren ihrer Mitarbeitenden auszumachen und damit nicht zuletzt einem der häufigsten Kündigungsgründe junger Nachwuchskräfte (vgl. Lancaster und Stillman 2010) entgegenzuwirken.

#### Arbeit als Berufung

Einige Menschen empfinden ihre Arbeit als Berufung, definiert als „verzehrende, bedeutungsvolle Leidenschaft, die Menschen für eine Domäne empfinden“ (Dobrow und Tosti-Kharas 2011, S. 1005). Menschen, die ihrer Arbeit als Berufung nachgehen, sind nachweislich erfolgreicher (Praskova et al. 2014), engagierter (Bunderson und Thompson 2009), stärker mit ihrem Unternehmen verbunden (Cardador et al. 2011) und erleben ihre Arbeit als sinnhafter (Hirschi 2011). Berufung bedeutet demnach, inspiriert und handlungsfähig in Übereinstimmung mit seinen wahren Potenzialen zu leben und zu arbeiten. Bloom et al. (2020) zeigten, dass, wenn Menschen mit einem starken Berufswunsch diesen auch ausüben können, sie im Laufe der Zeit ihre Berufung immer weiter stärken. Die Autoren beschreiben in der Entwicklung einer persönlichen Berufung zwei zentrale Faktoren: *Authentizität*, also wahrhaftig und kompetent für ihr ausgeübtes Handwerk zu stehen, und *Legitimität*, d. h., von ihrem Umfeld auch als fachliche Autorität anerkannt zu werden (Bloom et al. 2020, S. 32). Diese Aspekte können der sinnstiftenden Gestaltung von Nachwuchsprogrammen und Führungskräfteentwicklung dienen und auch für individuelle Zielvereinbarungsprozesse, Karrierepfade und Anreizsysteme genutzt werden. Dabei müssen Unternehmen jedoch darauf achten, dass sich Menschen mit einem starken Gefühl der Berufung durch ihr großes Engagement nicht selbst schaden. Duffy und Dik (2013) sehen neben den vielen positiven Effekten einer Berufung eine Gefahr darin, dass Menschen, die ihrer persönlichen Lebensaufgabe folgen, zu viel Energie in ihre Arbeit investieren und von ihren Arbeitgebern ausgebeutet werden könnten. Schabram und Maitlis (2017) weisen ebenfalls auf Burnout als potenzielle Schattenseite der Berufung hin.

### Team: Purpose als Konsens und soziale Bindung

#### Soziale Identität

Teams stellen eine Form der Gemeinschaft dar, aus deren Zugehörigkeit Menschen Bedeutung (Pratt und Ashford 2003) und intrinsische Motivation schöpfen (Gagné und Deci 2005) und daraus einige ihrer sozialen Identitäten definieren. Griffin (1983) geht davon aus, dass die Sinnhaftigkeit, die Mitarbeitende einer Arbeitsaufgabe beimessen, sozial konstruiert wird. Je mehr Wertschätzung eine Aufgabe durch Führungskräfte und Kollegen erfährt, umso sinnvoller wird sie wahrgenommen. Mitarbeitende suchen demnach an ihrem Arbeitsplatz nach Hinweisen, wie sie sich am vorteilhaftesten verhalten sollen, und konstruieren daraus ihre Einstellungen, Interpretationen und Bedeutungen der Arbeit. So entwickeln sich gemeinschaftliche Normen und Regeln (Wrzesniewski et al. 2003). Maitlis und Christianson (2014) unterstreichen das Bedürfnis nach Sinn hinter dem eigenen Handeln und dem des Umfeldes: Wenn Menschen auf Momente der Mehrdeutigkeit oder Ungewissheit stoßen, versuchen sie zu klären, was vor sich geht, indem sie Hinweise aus ihrer Umgebung wahrnehmen, interpretieren und so einen Sinn im Geschehen ausmachen, der ihr weiteres Handeln beeinflusst. Purpose, als Orientierungspunkt und schließende Handlungsstrategie (s. unten), kommt demnach auch eine sozial normierende und konsensbildende Funktion zu (Gebert et al. 2010).

#### Beziehungsqualität und prosoziale Arbeitsgestaltung

Die Beziehungen zu anderen Menschen sind für unsere Wahrnehmung von Sinn und Bedeutsamkeit maßgeblich. Robertson et al. (2020) skizzieren die Rolle sozialer Verbindungen für das Purpose-Erleben von Mitarbeitenden. Sie nehmen an, dass Ressourcen aus beruflichen Netzwerken, die hierarchisch geprägt bzw. informell sein oder auf Expertentum basieren können, das Maß beeinflussen, in dem ein Mensch seine Tätigkeit als sinnhaft empfindet. Solche Ressourcen können instrumenteller Natur sein, wie z. B. Informationen und Ratschläge für die Erledigung von Aufgaben, oder expressiver Natur, wie soziale Unterstützung, Mitgefühl und Freundschaft (Umphress et al. 2003). Auf diese Weise verhelfen Netzwerke Menschen dazu, sich integriert zu fühlen, ihre Aufgaben gut zu erledigen und neue Fähigkeiten zu erwerben. Die Digitalisierung und IT-gestützte Kommunikation bringen weitere Implikationen für soziale Verbindungen am Arbeitsplatz mit sich: So postulieren Wang et al. (2020), dass der Austausch instrumenteller Unterstützung in der digitalen Kommunikation eine stärkere Rolle spielt und so die Produktivität und Aufgabenerledigung steigert. Expressive Verbindungselemente wie Körpersprache und soziale Hinweisreize sind digital dagegen eingeschränkter abbildbar.

Wenn Unternehmen ihren Mitarbeitenden die Möglichkeit bieten, etwas Wertvolles für andere Mitglieder der Gemeinschaft beizutragen, gewinnen diese Mitarbeitenden ein Gefühl von Zweck, Handlungsfähigkeit und Einfluss (Grant 2007). Folglich können Organisationen der Arbeit auch Richtung und Intentionalität – also Purpose – verleihen, indem sie die Mitarbeitenden dabei fördern, starke soziale Bindungen in der Organisation aufzubauen. Gezielte Peer-Learning-Formate etwa, in denen Mitarbeitende von Mitarbeitenden lernen, umfassen instrumentelle und expressive Ressourcen gleichermaßen.

### Organisation: Purpose durch Führungsverhalten und Strukturen

#### Visionäre Führung und sinnbasiertes Führungsverhalten

Visionäre Führung und sinnbasiertes Führungsverhalten sind wie zwei Seiten einer Medaille. Führungskräfte haben die Aufgabe, die Unternehmensvision so zu vermitteln, dass deren Inhalt und Sinnhaftigkeit von allen Mitarbeitenden gleich verstanden wird. Eine Vision kann als ein lebendiges, verbalisiertes Idealbild dessen definiert werden, was die Organisation eines Tages erreichen möchte (Rafferty und Griffin 2004, S. 332). Die Kommunikation der Führungskräfte über verschiedene Ereignisse und Umstände beeinflussen das arbeitsbezogene Sinnempfinden der Menschen (Podolny et al. 2004). Dazu sollte laut Carton et al. (2014, S. 1547) die Vision in einer möglichst vorstellbaren Sprache beschrieben werden. Sie zeigten, dass es Führungskräften, die mit Bildsprache kommunizierten, besser gelang, ein übereinstimmendes Verständnis der Vision unter den Teammitgliedern zu erzeugen, als durch das Verwenden von abstrakter Konzeptrhetorik (Carton et al. 2014). Van Knippenberg (2020, S. 9) beschreibt dazu einen neuen Ansatz der sinnbasierten Führung. Sein Kerngedanke folgt der Logik, dass Führung, die für die Grundausrichtung der Organisation steht, einen entsprechenden gemeinsamen Sinn und Zweck im Team inspiriert und somit gruppenspezifisches Handeln motiviert, das wiederum auf diesen Sinn einzahlt. Er greift in seinen Ausführungen das Problem von Purpose auf (das ebenfalls für Vision und Mission gilt), dass dieser – auf der Ebene der Organisation – immer abstrakt formuliert werden muss, dadurch jedoch nicht zu konkreten Handlungen führen kann. Abteilungen bzw. Fachrichtungen in Unternehmen, denken wir beispielsweise einfach an produzierende vs. verwaltende Berufe, haben jeweils sehr unterschiedliche Handlungsweisen, mit welchen sie auf den Purpose eines Unternehmens einzahlen. Führungskräften kommt die Rolle zu, den abstrakt formulierten Purpose in konkrete Handlungsweisen auf Team- und Abteilungsebene zu übersetzen bzw. ihr Team zu befähigen, gemeinsam diese Übersetzungsarbeit zu leisten. Van Knippenberg (2020) betont hierzu, dass erst das ‚Empowerment‘ des Teams durch die Führungskraft zur gemeinsamen Konkretisierung des Purpose in Handlungen und zur gewünschten Verbindlichkeit und Verinnerlichung des Purpose führt. Der Erfolg der Führungskraft bei der Vermittlung des Purpose wird davon beeinflusst, wie stark diese persönlich mit dem Purpose in Verbindung gebracht wird (*Legitimität*), z. B. weil sie in der Unternehmenshierarchie eine lenkende (hohe) Funktion bekleidet oder weil die Führungskraft durch ihr Handeln als Verkörperung der organisatorischen Identität wahrgenommen wird (*Prototypizität*).

#### Strukturen und Freiheitsgrade

Im Kontext Führung wird aktuell viel über flache Hierarchien, Freiräume und Selbstorganisation gesprochen. Die Argumentation ist hier meist, dass starre Strukturen aufgebrochen werden müssen, um Innovation und Anpassung an Veränderungen zu ermöglichen. Zhang et al. (2015) beschreiben bei der Vorstellung ihres Konzepts des paradoxen Führungsverhaltens, wie scheinbar widersprüchliche und doch verbundene Verhaltensweisen von Führungskräften dazu führen, dass Teams konkurrierenden Anforderungen am Arbeitsplatz gerecht werden. Es zeigt sich, dass alternativ ein stabiler Rahmen geschaffen werden kann, der den Mitarbeitenden innerhalb festgesetzter Grenzen flexibles Handeln ermöglicht und ihnen gleichzeitig den Halt gibt, für den sonst die Führungskraft sorgen würde (Gebert et al. 2010). Dieses Phänomen erklärt beispielsweise den Erfolg agiler Methoden wie Scrum (Pichler 2009). Nicht *trotz* der Regeln funktionieren diese, sondern *weil* diese Regeln die angestrebte Agilität unterstützen, indem sie eine strukturierte und zugleich dynamische Routine gewährleisten und zu jedem Zeitpunkt sicherstellen, dass alle Teammitglieder wissen, was von ihnen erwartet wird. Ein eindeutiger Purpose bildet in ähnlicher Weise den Rahmen für das Handeln der Mitarbeitenden: Ein niederländisch-amerikanisches Team fand bei der Untersuchung selbstorganisierter Teams heraus, dass die Voraussetzung für deren Leistungsfähigkeit das Vorhandensein einer gemeinsamen Zielausrichtung ist (Nederveen Pieterse et al. 2019). Somit liegt es nahe, dass ein klarer Purpose als Voraussetzung für Selbstorganisation seinen Mehrwert im Sinne höherer Anpassungsfähigkeit und zielgerichteter Leistungsfähigkeit entfaltet. Carton et al. (2014, S. 1548) erklären weiterhin die Funktion einer gemeinsamen Ausrichtung wie folgt: Je heterogener die Zielorientierung und das Zielbild im Team sind, umso höher ist der Koordinierungsaufwand des Teams, auf ein gemeinsames Ziel hinzuarbeiten. Fokus und Energie für die eigentliche Aufgabe gehen verloren. Teams mit heterogenem Zielbild profitieren deshalb stärker von hierarchischer Führung, während ein geteiltes Zielbild selbstorganisiertes Arbeiten erleichtert – bis dahin, dass es Führung ersetzen kann (siehe auch Gebert et al. 2010).

Den Purpose einer Organisation zu entwickeln und zu kommunizieren, bedeutet entsprechend, einen klaren Rahmen zu setzen, was dem Zweck der Organisation dient und was nicht. Purpose als Fixpunkt zur Orientierung ermöglicht die Freiheitsgrade, die für Innovation notwendig sind.

## *Warum* nützt Purpose Unternehmen?

Purpose als klarer Unternehmenszweck nützt Unternehmen durch drei Funktionen: Purpose wirkt, im Sinne einer schließenden Handlungsstrategie der Organisationsentwicklung, richtungsgebend (direktiv), fördert kulturell Konsens und ermöglicht es, neu entwickelte Ideen in der Praxis umzusetzen (Gebert et al. 2010). Ein geteilter Purpose verringert den Koordinationsaufwand von Aufgaben und Aktivitäten in Teams und Organisationen und steigert so deren Leistungsfähigkeit (Carton et al. 2014). Purpose vermindert Komplexität, indem er die Anzahl der Handlungsoptionen eines Teams reduziert. Folglich kann Führung über eine kluge Kombination von Purpose und Regeln viel Freiheit in der konkreten Umsetzung von Aufgaben zulassen, ohne direktiv zu agieren. Die durch Purpose erzeugte abgesicherte Freiheit befähigt die Mitarbeitenden zu autonomen Handeln (d. h. Empowerment) (Birkinshaw 2003; Gebert et al. 2010). So entsteht ein Klima der psychologischen Sicherheit, das jederzeit kreativen Widerspruch und kritisches Hinterfragen des Status quo erlaubt (Edmondson 2020). Erst durch die Kombination dieses gesetzten Rahmens mit der daraus erwachsenden Offenheit ist es möglich, alternative Blickwinkel einzunehmen, neue Optionen zu berücksichtigen und so Entwicklung zu ermöglichen (Gebert et al. 2010). Das permanente Hinterfragen von als gegeben betrachteten Tatsachen in Kombination mit einer klaren Zielorientierung ist die Voraussetzung für Innovation.

Purpose dient folglich als Puffer für die Komplexität, in der Unternehmen zunehmend agieren, und als gemeinsame Klammer, welche die Fliehkräfte in Schach hält. Je stärker der Veränderungsdruck ist, desto wichtiger wird die puffernde Gegenwirkung des Purpose, nicht im Sinne eines Entweder-oder, sondern im Sinne einer sinnvollen Kombination öffnender und schließender Handlungsstrategien von Führung (Gebert et al. 2010). Das ist auch der Grund, weshalb Kühl (im Interview mit Backovic 2021) argumentiert, dass Purpose bei einer angespannten wirtschaftlichen Entwicklung in der Post-Pandemiezeit an Bedeutung verlieren könnte. Wenn unternehmerischer Druck zur Konzentration auf Effizienzkriterien führt und die Rückkehr zu transaktionaler Führung nach sich zieht, reduziert sich der Bedarf nach Purpose zwangsläufig. Oder anders ausgedrückt: Je instabiler die Umwelt eines Unternehmens wird, desto flexibler muss mit dem Purpose umgegangen werden.

Eine Organisation kann auch an einer zu starren Orientierung an einem Purpose zugrunde gehen. Ein Beispiel hierfür kann der Vergleich von Blockbuster und Netflix liefern. Während sich der damalige Marktführer Blockbuster als DVD-Vermietungs-Service definierte, sah sich das Start-up Netflix als Online-Movie-Service. Durch den anders gelagerten Purpose hat sich Netflix bereits frühzeitig auf sämtliche Formen, Filme an Zuschauer auszuliefern, fokussiert, während Blockbuster in seinem zunächst erfolgreichen Geschäftsmodell der DVD-Vermietung verharrte, bis das Unternehmen schließlich insolvent war (O’Reilly und Tushman 2016).

Der eigentliche Hebel für die Zukunftsausrichtung von Unternehmen besteht in der Analyse der strategischen Entwicklung, d. h. des Zusammenspiels von Stabilität und Veränderung, und die clevere Planung dazugehöriger Puffermaßnahmen. Organisationen, die die Antwort auf das *why* des WHY geben können, entwickeln ihren Purpose im skizzierten Sinne und profitieren von seiner Funktion.

## Diskussion

Im vorliegenden Beitrag wurde theoriegeleitet dargestellt, *was* Purpose bedeutet, *wie* Unternehmen ihren Purpose ihrem Organisationszweck entsprechend auf den drei Ebenen Individuum, Team und Organisation entwickeln können und *welchem Zweck* die Entwicklung und Einführung dieses Purpose dient. Damit wird Orientierung, Klarheit und Evidenz zur Diskussion rund um dieses wichtige, jedoch bisher unscharf behandelte Konstrukt beigetragen.

Wissenschaftlich gilt es, weitere Untersuchungen zur Validierung des Purpose-Konstruktes anzustellen. Im Bereich des individuellen Sinnempfindens fehlt es an Erkenntnissen, wie sich Purpose über die skizzierten Zusammenhänge hinaus im Zusammenspiel mit Team und Organisation gestaltet, beispielsweise anknüpfend an Studien zum Person-Organization-Fit (Kristof-Brown et al. 2005). Insgesamt fehlen auf den Ebenen Team und Organisation tiefergehende Studien. Das Modell sinnbasierter Führung von van Knippenberg (2020) sollte in Feldstudien und experimentellen Designs empirisch untersucht werden. Interessant wären auch vertiefte Studien zu Unterschieden hinsichtlich der Wahrnehmung von Purpose in For-Profit- versus Non-Profit-Organisationen (ebd.) ebenso wie eine Untersuchung der wachsenden Anzahl von Organisationen, die zwar einen kommerziellen Zweck verfolgen, sich aber dennoch stark ökologischen bzw. gesellschaftlich-sozialen Zwecken verschreiben. Ein weiteres lohnenswertes Forschungsfeld stellen die zunehmende digitale Arbeit, Zusammenarbeit in ortsverteilten Teams und die damit veränderte Beziehungsebene in Teams (Jiang 2021) und die zukünftige Rolle des Firmengebäudes als Arbeitsort dar. Die potenzielle Distanzierung von Mitarbeitenden von ihrer Firma und damit assoziierte schwindende Mitarbeiterbindung (Hinds und Cramton 2014), könnten durch einen starken Purpose kompensiert werden.

## Fazit

In Zeiten, in denen nicht nur durch globale Entwicklungen wie Digitalisierung oder Pandemien wie Covid-19 die Komplexität der Zusammenarbeit stetig steigt, brauchen Organisationen einen stabilen Faktor, an dem sie sich ausrichten können. Ursache-Wirkungsprinzipien sind vorab oft nicht klar erkennbar und auch erfahrene Organisationen müssen immer häufiger nach dem Prinzip Versuch und Irrtum handeln. Sie müssen daher ihre Arbeitsweisen in einem Umfeld von komplexer werdenden Märkten ändern, wenn sie weiterhin erfolgreich sein wollen. Die Komplexität erreicht einen großen Teil der Mitarbeitenden, die nun in den Blickpunkt rücken. Auch wenn es den Beispielen aus dem Chor der Influencer, die in nahezu allen Managementmagazinen zu lesen sind, an Definitionen, klar dargestellten Ursache-Wirkungs-Prinzipien und echter Empirie mangelt, sollten diese Schwächen Organisationen nicht davon abhalten, sich fundiert mit dem Thema Purpose zu befassen. Er erfüllt in dynamischen und sich stark verändernden Umgebungen – also unserer heutigen Arbeitswelt – eine enorm wichtige Funktion: die Komplexität der Handlungsoptionen zu reduzieren, indem er dem Handeln von Organisationen eine klare und idealerweise von allen Mitarbeitende verstandene und geteilte Grundausrichtung gibt. Dies ist der eigentliche Sinn von Purpose.

## Footnotes

1 „Sieben Kategorien von Mechanismen, die nach Ansicht von Wissenschaftlern dazu führen, dass Arbeit als sinnvoll wahrgenommen wird oder Bedeutung erlangt: Authentizität, Selbstwirksamkeit, Selbstwertgefühl, Zweckmäßigkeit, Zugehörigkeit, Transzendenz sowie kulturelle und zwischenmenschliche Sinnstiftung“ (Rosso et al. 2010, S. 108).

## References

[CR1] Backovic, L. (2021). Stefan Kühl im Interview: In ein, zwei Jahren wird kaum noch jemand über Purpose sprechen. *Handelsblatt online*, 4.https://www.handelsblatt.com/karriere/stefan--kuehl--im--interview--organisationsexperte--in--ein--zwei--jahren--wird--kaum--noch--jemand--ueber--purpose--sprechen/26238316.html. Zugegriffen: 14. Dezember 2021.

[CR2] Barrick MR, Mount MK, Li N (2013). The theory of purposeful work behavior: the role of personality, higher-order goals, and job characteristics. Academy of Management Review.

[CR3] Baumeister RF (1991). Meanings of life.

[CR4] Baumeister RF, Vohs KD, Snyder CR, Lopez SJ (2002). The pursuit of meaningfulness in life. Handbook of positive psychology.

[CR5] Birkinshaw, J. (2003). The Paradox of Corporate Entrepreneurship. *Strategy+business*. https://www.strategy-business.com/article/8276. Zugegriffen: 14. Dezember 2021.

[CR6] Bloom M, Colbert AE, Nielsen JD (2020). Stories of calling: how called professionals construct narrative identities. Administrative Science Quarterly.

[CR7] Bunderson JS, Thompson JA (2009). The call of the wild: Zookeepers, callings, and the double-edged sword of deeply meaningful work. Administrative Science Quarterly.

[CR8] Cardador MT, Dane E, Pratt MG (2011). Linking calling orientations to organizational attachment via organizational instrumentality. Journal of Vocational Behavior.

[CR9] Carton AM, Murphy C, Clark JR (2014). A (blurry) vision of the future: how leader rhetoric about ultimate goals influences performance. Academy of Management Journal.

[CR10] Dobrow SR, Heller D (2015). Follow your heart or your head? A longitudinal study of the facilitating role of calling and ability in the pursuit of a challenging career. Journal of Applied Psychology.

[CR11] Dobrow SR, Tosti-Kharas J (2011). Calling: the development of a scale measure. Personnel Psychology.

[CR12] Duffy RD, Dik BJ (2013). Research on calling: What have we learned and where are we going?. Journal of Vocational Behavior.

[CR13] Edmondson AC (2020). Die angstfreie Organisation: Wie Sie psychologische Sicherheit am Arbeitsplatz für mehr Entwicklung, Lernen und Innovation schaffen.

[CR14] Frankl VE (1959). Man’s search for meaning.

[CR15] Gagné M, Deci EL (2005). Self-determination theory and work motivation. Journal of Organizational Behavior.

[CR16] Gebert D, Boerner S, Kearney E (2010). Fostering team innovation: why is it important to combine opposing action strategies?. Organization Science.

[CR17] Grant AM (2007). Relational job design and the motivation to make a prosocial difference. Academy of Management Review.

[CR18] Grant AM (2008). The significance of task significance: job performance effects, relational mechanisms, and boundary conditions. Journal of Applied Psychology.

[CR19] Griffin RW (1983). Objective and social sources of information in task redesign: a field experiment. Administrative Science Quarterly.

[CR20] Hinds PJ, Cramton CD (2014). Situated coworker familiarity: how site visits transform relationships among distributed workers. Organization Science.

[CR21] Hirschi A (2011). Callings in career: a typological approach to essential and optional components. Journal of Vocational Behavior.

[CR22] Jiang WY (2021). Sustaining meaningful work in a crisis: adopting and conveying a situational purpose. Administrative Science Quarterly.

[CR42] van Knippenberg D (2020). Meaning-based leadership. Organizational Psychology Review.

[CR23] Kristof-Brown AL, Zimmerman RD, Johnson EC (2005). Consequences of individuals’ fit at work: a meta-analysis of person-job, person-organization, person-group, and person supervisor fit. Personnel Psychology.

[CR24] Lackner K, Schuster M (2020). Sinn, Zweck und Purpose im Organisationskontext: Magic Moments. Gruppe. Interaktion. Organisation. Zeitschrift für Angewandte Organisationspsychologie (GIO).

[CR25] Lancaster LC, Stillman D (2010). The mindfactor: how the millennial generation is rocking the workplace.

[CR26] Maitlis S, Christianson M (2014). Sensemaking in organizations: taking stock and moving forward. Academy of Management Annals.

[CR27] Nederveen Pieterse A, Hollenbeck JR, van Knippenberg D, Spitzmüller M, Dimotakis N, Karam EP, Sleesman DJ (2019). Hierarchical leadership versus self-management in teams: goal orientation diversity as moderator of their relative effectiveness. The Leadership Quarterly.

[CR28] O’Reilly CA, Tushman M (2016). Lead and disrupt: How to solve the innovator’s dilemma.

[CR29] Pichler R (2009). Scrum – Agiles Projektmanagement erfolgreich einsetzen.

[CR30] Podolny JM, Khurana R, Hill-Popper M (2004). Revisiting the meaning of leadership. Research in Organizational Behavior.

[CR31] Praskova A, Hood M, Creed PA (2014). Testing a calling model of psychological career success in Australian young adults: a longitudinal study. Journal of Vocational Behavior.

[CR32] Pratt MG, Ashford BE, Cameron KS, Dutton JE, Quinn RE (2003). Fostering meaningfulness in working and at work. Positive organizational scholarship: foundations of a new discipline.

[CR33] Rafferty AE, Griffin MA (2004). Dimensions of transformational leadership: conceptual and empirical extensions. The Leadership Quarterly.

[CR34] Robertson KM, O’Reilly J, Hannah DR (2020). Finding meaning in relationships: the impact of network ties and structure on the meaningfulness of work. Academy of Management Review.

[CR35] Rosso BD, Dekas KH, Wrzesniewski A (2010). On the meaning of work: a theoretical integration and review. Research in Organizational Behavior.

[CR36] Ryff CD (1989). Happiness is everything, or is it? Explorations on the meaning of psychological well-being. Journal of Personality and Social Psychology.

[CR37] Ryff CD (1989). Beyond ponce de Leon and life satisfaction: new directions in quest of successful ageing. International Journal of Behavioral Development.

[CR38] Schabram K, Maitlis S (2017). Negotiating the challenges of a calling: emotion and enacted sensemaking in animal shelter work. Academy of Management Journal.

[CR39] Schwartz SH (1992). Universals in the content and structure of values: theoretical advances and empirical tests in 20 countries. Advances in Experimental Social Psychology.

[CR47] Selznick, P. & Carlston, K. S. (1957). Law and the Structures of Social Action. Administrative Science Quarterly, 2(2), 258. 10.2307/2390698

[CR40] Sinek S (2019). Start with why: how great leaders inspire everyone to take action.

[CR41] Umphress EE, Labianca G, Brass DJ, Kass E, Scholten L (2003). The role of instrumental and expressive social ties in employees’ perceptions of organizational justice. Organization Science.

[CR43] Wang B, Liu Y, Parker SK (2020). How does the use of information communication technology affect individuals? A work design perspective. Academy of Management Annals.

[CR44] Williamson Smith R, DeNunzio MM (2020). Examining personality—job characteristic interactions in explaining work outcomes. Journal of Research in Personality.

[CR45] Wrzesniewski A, Dutton JE, Debebe G (2003). Interpersonal sensemaking and the meaning of work. Research in Organizational Behavior.

[CR46] Zhang Y, Waldman DA, Han Y-L, Li X-B (2015). Paradoxical Leader Behaviors in People Management: Antecedents and Consequences. Academy of Management Journal.

